# Soft Tissue Stability around Single Implants Inserted to Replace Maxillary Lateral Incisors: A 3D Evaluation

**DOI:** 10.1155/2016/9393219

**Published:** 2016-05-19

**Authors:** F. G. Mangano, F. Luongo, G. Picciocchi, C. Mortellaro, K. B. Park, C. Mangano

**Affiliations:** ^1^Department of Surgical and Morphological Science, Dental School, University of Varese, 21100 Varese, Italy; ^2^Private Practice, 00193 Rome, Italy; ^3^Private Practice, 20154 Milan, Italy; ^4^Department of Health Sciences, University of Eastern Piedmont, 28100 Novara, Italy; ^5^Mir Dental Hospital, 149-132 Samduk 2Ga, Jung-Gu, Daegu 700 412, Republic of Korea; ^6^Department of Dental Sciences, Vita Salute San Raffaele University, 20132 Milan, Italy

## Abstract

*Purpose*. To evaluate the soft tissue stability around single implants inserted to replace maxillary lateral incisors, using an innovative 3D method.* Methods*. We have used reverse-engineering software for the superimposition of 3D surface models of the dentogingival structures, obtained from intraoral scans of the same patients taken at the delivery of the final crown (S1) and 2 years later (S2). The assessment of soft tissues changes was performed via calculation of the Euclidean surface distances between the 3D models, after the superimposition of S2 on S1; colour maps were used for quantification of changes.* Results*. Twenty patients (8 males, 12 females) were selected, 10 with a failing/nonrestorable lateral incisor (*test* group: immediate placement in postextraction socket) and 10 with a missing lateral incisor (*control* group: conventional placement in healed ridge). Each patient received one immediately loaded implant (Anyridge®, Megagen, Gyeongbuk, South Korea). The superimposition of the 3D surface models taken at different times (S2 over S1) revealed a mean (±SD) reduction of 0.057 mm (±0.025) and 0.037 mm (±0.020) for* test* and* control* patients, respectively. This difference was not statistically significant (*p* = 0.069).* Conclusions*. The superimposition of the 3D surface models revealed an excellent peri-implant soft tissue stability in both groups of patients, with minimal changes registered along time.

## 1. Introduction

In recent years, aesthetics has become increasingly important: everyone wants to be beautiful, according to modern society's concept of beauty. Since a beautiful smile can make the difference, dental and oral implantology are no exception: patients have high aesthetic expectations of implant-prosthetic treatment and require cosmetic restorations that are indistinguishable from natural teeth [1, 2]. This is why the reconstruction of single missing teeth in the aesthetic areas of the maxilla with dental implants is currently a challenge for the surgeon and the prosthodontist [2–4].

The loss of a tooth actually results in a contraction of the hard and soft tissues [5–7]. The gradual involution and reduction of alveolar bone volume begin immediately after extraction and are accompanied by a contraction of the overlying soft tissues [6–8]. As demonstrated in various animal [9–12] and human [13–16] studies, a major contraction of the alveolar bone volume occurs after extraction of natural teeth, during the first six months and up to 2 years after extraction. A marked reduction in the buccopalatal width and the height of the alveolar ridge is already evident in the first year after the extraction [7, 8, 14] and is accompanied by a contraction of the overlying soft tissues [6, 8, 17, 18]. These phenomena are particularly evident in the anterior maxilla [7, 8, 14, 16], since in this area tooth extraction compromises the vascularization of the delicate vestibular bone plate, mainly provided by the vascular plexus of the periodontal ligament. The immediate consequence of reduced vascularization in the anterior maxilla is the physiological horizontal and vertical resorption of the vestibular bone plate, with contraction of the overlying soft tissues [7, 8, 14, 17, 18]. This contraction can make implant-prosthetic treatment unpredictable when restoring and maintaining a cosmetic appearance identical to that of a natural tooth are our purpose [17, 18].

The long-term stability of the peri-implant soft tissues in the anterior maxilla is of fundamental importance for the success of implant treatment [17, 18]. For this reason, over the years, a whole series of indexes for describing the aesthetic outcome of implants and for monitoring the stability of the peri-implant soft tissues over time have been proposed in the literature [19–24]. Although these indexes, particularly some of them [21, 22], have been used by various authors [2, 4, 25–27] and have represented the standard for evaluating the aesthetic success of reconstructions with single implants in the anterior maxilla [21, 22, 25–27], until now it was not possible to perform an exact quantitative evaluation of the stability of the peri-implant soft tissues over time [28]. In fact, the indexes proposed in the literature are based on a two-dimensional photographic assessment (2D) and on the comparison of photographs taken at different times (usually at the delivery of the final restoration and over the following years) through the application of established criteria [28]. This does not allow the actual loss or the three-dimensional contraction (3D) of the peri-implant tissues over time to be exactly quantified [28]. To date, only one clinical work has tried to quantitatively assess the modifications of the soft tissues around individual implants in the aesthetic area over time [29]; however, this study employed reconstructions from cone beam computed tomography (CBCT), which is not ideal for the purpose.

Various surgical techniques have been proposed for placing of implants in the anterior maxilla [4, 25–27]. Amongst them, we should mention the immediate placement of implants in postextraction sockets, early implant placement in sites where bone healing is still ongoing (4–8 weeks after extraction), and conventional implant placement in fully healed sites (6–8 months after extraction) [4, 25–27]. Although all of these techniques can provide high implant survival, it is not yet clear which of these may give the best aesthetic result [4, 25–28].

Recent advances in the field of digital dentistry, and in particular the introduction of intraoral scanners which are powerful devices for taking optical impressions [30, 31], could help clinicians to fully understand the dynamics and transformations of the peri-implant soft tissue over time: this is of particular interest for single implants positioned in the anterior maxilla. In fact, the patient can undergo various scans with the intraoral scanner in the course of the treatment (e.g., at the time of placement of the final restoration and during subsequent check-ups). The patient's 3D models can then be loaded into reverse-engineering software and superimposed over each other [32, 33], in order to exactly quantify the stability of the peri-implant soft tissues over time.

Hence, the aim of this study was to compare the stability of peri-implant soft tissues around single implants positioned in the anterior maxilla, over time, with two different surgical protocols (immediate implants versus conventional implants), using an innovative 3D technique.

## 2. Materials and Methods

### 2.1. Study Population

The present study was designed as a prospective investigation based on data from patients recruited/treated in two different private practices (Gravedona, Como and Rome, Italy), under a standardized protocol, over a two-year period (September 2011–2013).

Inclusion criteria were patients in good oral/systemic health, in need for replacement of failing/nonrestorable maxillary lateral incisors (immediate implants in postextraction sockets:* test* group) or missing lateral incisors (conventional implants in healed ridges:* control* group), with sufficient bone height/width to place an implant of at least 3.5 mm in diameter and 10.0 mm in length, and with natural teeth adjacent to the implant site. In the* control* group, both patients with congenitally missing lateral incisors and patients who had previously lost a lateral incisor (with at least 4 months of healing after tooth extraction) were included.

Exclusion criteria were patients with active oral infections, chronic periodontitis with advanced loss of support (defined by periodontal pocking depth > 6 mm with clinical attachment loss > 4 mm, radiographic evidence of bone loss and increased tooth mobility), and patients with severe systemic diseases that would not allow a surgical intervention (immunocompromised patients, patients who underwent radiotherapy and/or chemotherapy, and patients in treatment with intravenous and/or oral amino-bisphosphonates). Smoking was not an exclusion criterion, although all patients were informed that smoking is associated with an increased risk of implant failure [34]. All patients received full explanation about the surgical and prosthetic protocol and signed an informed consent form prior to being enrolled in the present study; all patients accepted to fully participate in surveys. The Ethics Committee for Human Studies of the Hospital of Varese approved the present study, which was conducted in accordance with the principles outlined in the Declaration of Helsinki of 1975, as revised in 2008.

### 2.2. Surgical and Prosthetic Protocol

The surgical and prosthetic protocol was as previously reported [35]. In brief, all patients received one single implant (Anyridge, Megagen, Gyeongbuk, South Korea), placed to replace a failing/nonrestorable or a missing maxillary lateral incisor.

The fixtures used in the present have a tapered design with aggressive threads and a calcium-incorporated nanostructured surface (Xpeed®, Megagen, Gyeongbuk, South Korea) with the potential to accelerate healing processes and to promote osseointegration [36]. In addition, they have a conical connection (10°), which offers a tight seal and a built-in platform switching, ideal for preventing crestal bone resorption and for maintaining soft tissue volume along time [35].

Prior to surgery, a careful preoperative clinical and radiographic assessment was made in each patient, with the aim of better understanding the anatomy of implant sites; moreover, impressions were taken, casts were developed, and a diagnostic wax-up was performed, in order to better understand the patient's prosthetic needs.

In postextraction sockets, after local anaesthesia, an intrasulcular incision was made, extended to the neighboring teeth, and the failing/nonrestorable tooth was gently extracted, avoiding any movement that could damage the buccal bone wall. The postextraction socket was carefully debrided and the integrity of the socket walls was checked. After that, the surgical site was prepared, by deepening the socket for 3-4 mm, and the implant was placed; finally, particles of synthetic bone grafts were used to fill the gap between the implant body and the buccal bone wall and to overbuild the buccal bone wall, for protection against bone resorption.

In healed sites, after local anaesthesia, a crestal incision was made, connected with two lateral (vertical) releasing incisions, and a full-thickness surgical flap was raised to expose the alveolar crest; then, the surgical site was prepared according to the manufacturer's recommendations, and the implant was placed.

The surgeons were free to choose between different implant lengths (10.0 mm, 11.5 mm, and 13.0 mm) and diameters (3.5 mm and 4.0 mm). In both postextraction sockets and healed sites, the implants were placed slightly palatally, in order to avoid contact with the buccal bone wall. The implant stability was checked manually at placement. Sutures were placed. All implants were functionally loaded immediately after placement, with a provisional crown. Patients were prescribed with oral antibiotics (amoxicillin plus clavulanic acid, 2 gr/day for 6 days) and analgesics (ibuprofen, 600 mg/day for 3 days). Ice packs were provided, and a soft diet was recommended for the first week. After one week, sutures were removed.

The provisional crown remained* in situ* for a period of 3 months; after that final impressions were taken and the final metal-ceramic crown was provided. All crowns were cemented with a temporary zinc-eugenol cement. All patients were enrolled in a 6-month postoperative control program.

### 2.3. Intraoral Scans

Each patient underwent two different intraoral scans of the full mouth: three months after implant placement, at the delivery of the final implant-supported restoration (S1), and two years later (S2). All scans were performed by two calibrated operators, with proven experience in the use of intraoral scanners. All scans were performed with a powerful, modern intraoral scanner (Trios, 3-Shape, Copenhagen, Denmark). This structured-light device works under the principle of confocal microscopy and ultrafast optical scanning, and it produces in-color 3D surface models in a proprietary (.DCM) format [33]. These files were then converted into solid-to-layer (.STL) files, using proprietary software.

### 2.4. 3D Soft Tissues Evaluation

The 3D surface models of the two different scans (S1 and S2) from each patient were imported into powerful reverse-engineering software (Geomagic Studio 2012, Geomagic, Morrisville, NC, USA) [36]. All scans were checked and cleaned using the “mesh doctor” function, so that small artifacts identified as independent polygons could be automatically removed. After that, the scans were cut and trimmed using the “cut with planes” function, in order to obtain uniform surface models, representing the implant-supported restoration and the adjacent natural teeth only, with related soft tissues. Subsequently, the function “cut with lines” was used to isolate the soft tissues from the implant-supported restoration and the adjacent natural teeth. The obtained uniform 3D surface models represented the region of interest for this study: they were saved in specific folders and were ready for superimposition. Superimposition was obtained as follows. First, the S2 model was roughly superimposed to the S1 model (reference dataset) using the “three-point” registration tool. The three points were identified on the final implant-supported crown, two on the crown margin and one on the cervical area, in order to facilitate this alignment. After this first rough alignment, the final registration was performed using the “best fit alignment” function. This final registration was obtained using an iterative closest point algorithm, also called “robust iterative closest point” (RICP). The distances between the S1 and the S2 models were minimized using the point-to-plane method. For each case, approximately 65.000 triangles were superimposed. Congruence between specific corresponding structures was calculated at this stage, for testing the accuracy of the procedure. Finally, the distances between corresponding areas of S1 and S2 were color-coded on the superimposed models for visualization of the results; a color map was generated, where the distances between specific points of interest were quantified overall and in all three planes of space. The modifications of peri-implant soft tissues along time were therefore visualized and calculated as mean (± standard deviations, SD). The color maps indicated inward (blue) or outward (orange, red) displacement between overlaid structures, while an absence of changes was indicated by the green color. The analysis was repeated with four different settings (50 *μ*m, 25 *μ*m, 10 *μ*m, and 5 *μ*m), in order to help the reader to highlight the 3D deviations at different resolution/magnification. With the first two settings (50 *μ*m and 25 *μ*m), in fact, only the biggest variations affecting the tissues (>50 and >25 micrometers, resp.) could be visualized; with the last two (10 *μ*m and 5 *μ*m), it was possible to visually assess even little tissue variations along time (variations >10 and >5 micrometers, resp.). All the aforementioned procedures for 3D soft tissue evaluation along time were made by the same calibrated operator, with extensive experience with reverse-engineering software and software for overlapping of digital images.

### 2.5. Statistical Analysis

All collected data were inserted in a sheet for statistical analysis (Excel 2003®, Microsoft, Redmond, WA, USA). Mean ± SD of the modifications of peri-implant soft tissues along time were calculated for each patient and then for each group of patients (*test* versus* control* patients). The *t*-test for independent samples was used to evaluate the differences between the two groups. The level of significance was set at 0.05. All computations were carried out with statistical analysis software (SPSS 17.0®, SPSS Inc., Chicago, IL, USA).

## 3. Results

**Figure 1:**
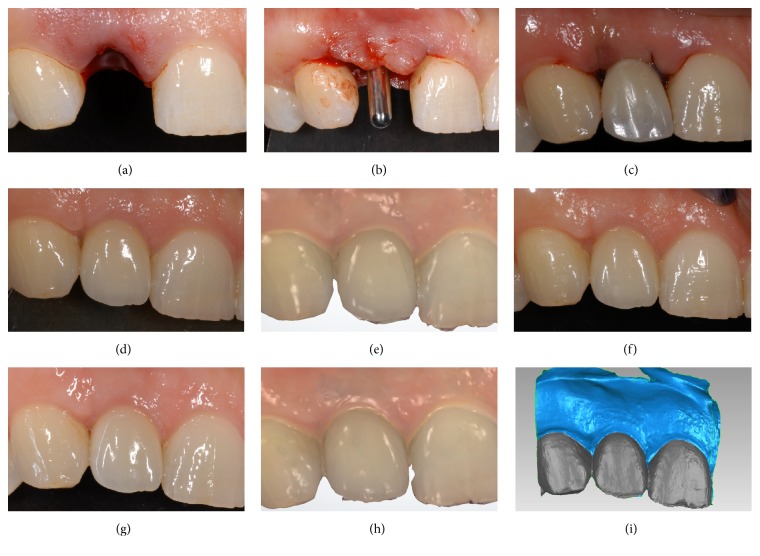
Immediate implant placement in postextraction socket (*test* group) of an adult female patient (34 years old): (a) the socket immediately after extraction; (b) the implant (Anyridge, Megagen, Gyeongbuk, South Korea) was placed in the fresh extraction socket; (c) the implant was immediately loaded with a provisional resin crown; (d) three months later, the final metal-ceramic crown was delivered to the patient; (e) first scan (S1) of the peri-implant soft tissues with a powerful intraoral scanner (Trios®, 3-Shape, Copenhagen, Denmark), at the delivery of the final crown; (f) 1-year clinical control; (g) 2-year clinical control; (h) second scan (S2) of the peri-implant soft tissues 2 years after the delivery of the final crown; (i) overlapping of digital images (S2 over S1) in powerful reverse-engineering software (Geomagic Studio 2012®, Geomagic, Morrisville, NC, USA).

**Figure 2:**
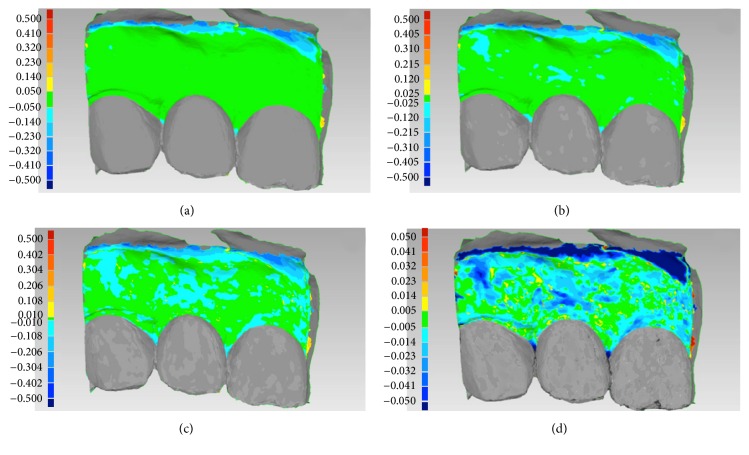
Immediate implant placement in postextraction socket (*test* group) of an adult female patient (34 years old): (a) overlapping of digital images (S2 over S1): colorimetric map, first setting (50 *μ*m). Since the variations in soft tissue volume over 2 years did not exceed 50 *μ*m, the only color visualized was green; (b) overlapping of digital images (S2 over S1): colorimetric map, second setting (25 *μ*m). Only in a few restricted areas was a variation/reduction in soft tissue volume > 25 *μ*m registered: therefore, the predominant color was still green; (c) overlapping of digital images (S2 over S1): colorimetric map, third setting (10 *μ*m). Overall, the soft tissues were stable and did not show contractions > 10 *μ*m, but the soft tissues overlying the vestibular (bundle) bone showed some kind of variation/reduction over time, as they were depicted in light blue; (d) overlapping of digital images (S2 over S1): colorimetric map, fourth setting (5 *μ*m). The area of the vestibular mucosa overlying the vestibular (bundle) bone was clearly the most affected by tissue contraction over time, although the mean (±SD) soft tissue reduction in the whole inspected area amounted to 0.024 mm (±0.048) only.

**Figure 3:**
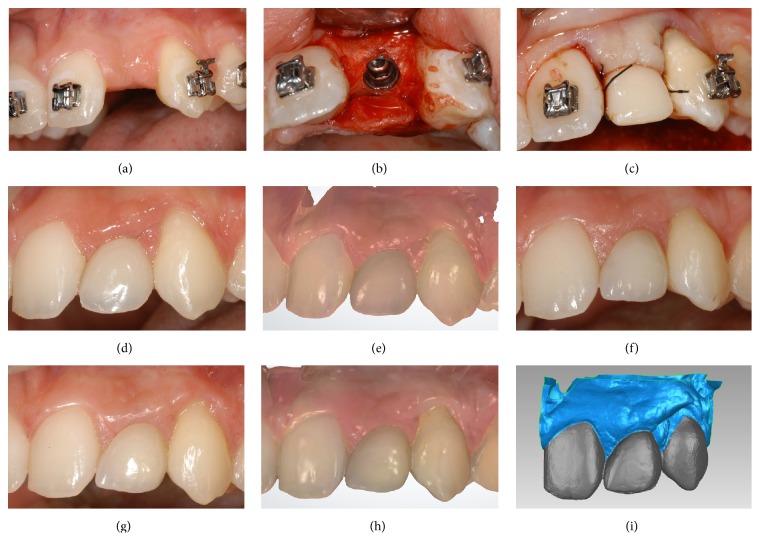
Conventional implant placement in healed ridge (*control* group) of a young female patient (19 years old) who underwent orthodontic treatment: (a) preoperative situation; (b) the mucoperiosteal flap was raised, the alveolar bone was exposed and the implant (Anyridge, Megagen, Gyeongbuk, South Korea) was placed in the healed ridge; (c) the implant was immediately loaded with a provisional resin crown; (d) three months later, the final metal-ceramic crown was delivered to the patient; (e) first scan (S1) of the peri-implant soft tissues with a powerful intraoral scanner (Trios, 3-Shape, Copenhagen, Denmark), at the delivery of the final crown; (f) 1-year clinical control; (g) 2-year clinical control; (h) second scan (S2) of the peri-implant soft tissues 2 years after the delivery of the final crown; (i) overlapping of digital images (S2 over S1) in powerful reverse-engineering software (Geomagic Studio 2012, Geomagic, Morrisville, NC, USA).

**Figure 4:**
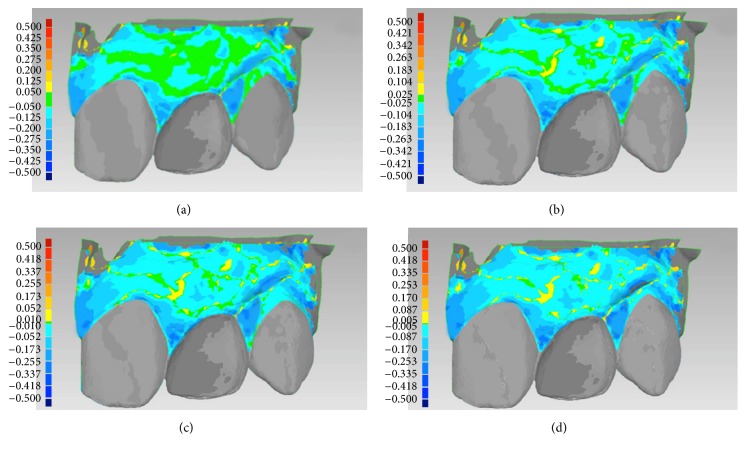
Conventional implant placement in healed ridge (*control* group) of a young female patient (19 years old) who underwent orthodontic treatment: (a) overlapping of digital images (S2 over S1): colorimetric map, first setting (50 *μ*m). The soft tissues overlying the vestibular (bundle) bone appeared stable, while the papillae showed some kind of contraction; however, this could be related to the movements of the natural teeth adjacent to the implant-supported restoration; (b) overlapping of digital images (S2 over S1): colorimetric map, second setting (25 *μ*m). The predominant color was light blue, since in most areas a variation/reduction in soft tissue volume > 25 *μ*m was registered; (c) overlapping of digital images (S2 over S1): colorimetric map, third setting (10 *μ*m). Only a few areas showed contraction < 10 *μ*m; (d) overlapping of digital images (S2 over S1): colorimetric map, fourth setting (5 *μ*m). The area of the vestibular mucosa overlying the vestibular (bundle) bone was the least affected by tissue contraction over time, whereas the papillae were the most affected. Overall, the mean (±SD) soft tissue contraction/reduction in the whole inspected area amounted to 0.091 mm (±0.073).

Six patients did not match the inclusion criteria and were therefore excluded from the study. Twenty patients (8 males, 12 females; aged between 17 and 54 years) with failing/nonrestorable or missing lateral incisors presented no conditions enlisted in the exclusion criteria and were enrolled in the present study. Ten patients (5 males, 5 females; aged between 19 and 54) had a failing/nonrestorable lateral incisor and were subjected to immediate implant placement (*test *group); among these patients, root fracture was the most frequent reason for tooth loss (5 patients), followed by caries (3 patients) and recurrent nontreatable endodontic lesions (2 patients). The other 10 patients (5 males, 5 females; aged between 17 and 34 years) had a missing lateral incisor (8 of them congenitally) and were therefore subjected to conventional implant placement (*control* group). Each patient received one single implant. All implants were functionally loaded immediately after placement. All implant-supported restorations were followed up for a period of 2 years after delivery (Figures 1 and 3). The superimposition of the 3D surface models taken at different times (S2 on S1) revealed a mean (±SD) reduction of 0.057 mm (±0.025) and 0.037 mm (±0.020) for* test* and* control* patients, respectively (Table 1, Figures 2 and 4). This difference was not statistically significant (*p* = 0.069). The changes evidenced between S1 and S2 were minimal, so that an excellent 3D peri-implant soft tissue stability along time was found in both groups of patients.

## 4. Discussion

Currently, the placement of single implants in the aesthetic area of the anterior maxilla is a difficult challenge for the surgeon and the prosthodontist [2–4]. On the one hand, in a world where a beautiful smile is becoming increasingly important, the patient's aesthetic expectations are in fact higher than ever [2, 4]; on the other, it is known that the loss of a tooth inevitably results in resorption of alveolar bone, with consequent contraction of the overlying soft tissues [5–7].

A recent systematic review on clinical studies by Tan and colleagues [7] has confirmed that, after tooth extraction, a pronounced horizontal dimensional reduction (3.79 ± 0.23 mm) combined with a vertical reduction (1.24 ± 0.11 mm on buccal, 0.84 ± 0.62 mm on mesial and 0.80 ± 0.71 mm on distal sites) occurs at 6 months; percentage horizontal and vertical dimensional changes were comprised between 29–63% and 11–22% at 6 months, respectively [7]. The amount of bone resorption is usually greater at the buccal aspect than at its palatal/lingual counterpart, particularly in the anterior maxilla [7, 8, 13, 15, 16]. In fact, most tooth sites in the anterior maxilla exhibit very thin (≤1 mm) buccal bone walls that are frequently made up of only bundle bone [13, 15, 16, 37, 38]. As the bundle bone is a tooth-dependent structure, such a thin bone wall may undergo marked resorption following tooth extraction [37, 38]. Chappuis and colleagues have identified a buccal bone wall thickness of ≤1 mm as a critical factor associated with the extent of bone resorption [14]. Thin-wall phenotypes displayed pronounced vertical bone resorption, with a median bone loss of 7.5 mm, as compared with thick-wall phenotypes, which decreased by only 1.1 mm [14].

Various treatment modalities have been described for implant therapy in the anterior zone such as conventional (4–6 months after tooth extraction), early (typically 4–8 weeks after extraction), and immediate implant placement (placement of a dental implant at the time of tooth extraction) [1, 2, 4, 25, 26]. Immediate implant placement has several advantages over the other treatment modalities, since it reduces the number of dental appointments, the time of treatment, and the number of surgeries, improving patient acceptance, with the psychological benefit of simultaneously replacing a lost tooth with an implant [4, 25, 26].

However, it is not yet clear which of these techniques will ensure the best aesthetic results in the anterior maxilla [1, 2, 4, 25–28]. In fact, few studies have compared the aesthetic outcome of these different therapies and consequently the stability over time of the soft tissues around single implants placed in the aesthetic areas using the different surgical protocols mentioned above [4, 25–28].

In addition, almost all of these studies were based on 2D evaluation of photographs taken at different times during the course of therapy (usually at the time of delivery of the final restoration and at the time of subsequent follow-ups) [2, 4, 25–28]. In fact, the criteria introduced so far for evaluating the cosmetic success of the placement of single implants in the anterior maxilla are only 2D [19–24, 28]. Though these criteria can be useful for determining whether an implant-prosthetic restoration is cosmetically acceptable, they do not allow us to quantify changes in the peri-implant soft tissues over time [28, 29].

In order to quantify these changes with certainty, we must in fact have 3D models, obtained at different times during the course of therapy, so that we can overlay them with each other [29]. In this sense, the digital revolution, by introducing a series of powerful tools for capturing 3D images (cone beam computed tomography-CBCT, intraoral, extraoral, and face scanners) and reverse-engineering software for the processing/superimposition of images, can be of help [29–33].

In the last few years, various methods have been described for superimposition of 3D datasets, including landmark-based superimposition, surface-based superimposition, or voxel-based superimposition of form-stable anatomical structures [32, 33]. The validity of the first two superimposition techniques depends on the accuracy of landmark identification and on the precision of the 3D surface models, respectively [32]. The recent study by Chappuis and colleagues was the first ever to propose a technique for the 3D evaluation of the stability over time of the soft tissues around single implants placed in the anterior maxilla [29]. For this paper, the authors used a voxel-based overlay technique, reconstructing the peri-implant soft tissues from CBCT images [29]. Although this overlay method is safe and effective, the need of several CBCT scans, with consequent exposure to ionizing radiations, represents a major limitation of the procedure [29].

The introduction of the intraoral scanners, powerful tools for taking an optical impression [30], allows these problems to be overcome. Intraoral scanners actually allow us to obtain highly accurate 3D models of dentoalveolar tissue, using only a beam of light [30–33]. The scans can therefore be repeated at different times, without harming the patient. The purpose of the present prospective clinical study was to investigate the 3D stability of peri-implant soft tissues along time, in patients treated with a single implant for replacement of a maxillary lateral incisor. In order to quantitatively evaluate the 3D soft tissues dynamics, we have superimposed  .STL files of intraoral scans taken at different time (at the delivery of the final restoration, S1; and 2 years later, S2), using powerful reverse-engineering software. With this software, the 3D differences of the superimposed models (S2 on S1) were quantified and translated into color codes, representing the distance between corresponding points. Ten patients with a failing/nonrestorable lateral incisor (*test *group) and 10 with a missing lateral incisor (*control *group) were selected for the present study. Each patient received one single, immediately loaded implant. The final crowns were provided 3 months after surgery and monitored for a period of 2 years. At the end of the study, a mean loss of tissue of 0.057 mm (±0.025) and 0.037 mm (±0.020) was reported for* test* and* control* patients, respectively. This difference was not statistically significant (*p* = 0.069). The changes evidenced between S1 and S2 were minimal, so that an excellent 3D peri-implant soft tissue stability along time was found. In general, the contraction of the tissues mostly affected the vestibular mucosa over the implant, as expected; this decrease was more pronounced in the case of immediate implants (*test* group); implants placed in healed ridges (*control* group) showed a lesser modification in this area and major changes in the papillae. The overall best results obtained in the present study with immediate implants (*test* group) may be in some way related to the use of bone grafting material for the protection of the buccal bone. However, these issues are worthy of further investigation and analysis: in fact, factors affecting soft tissue level around anterior maxillary single-tooth implants still need to be elucidated [39].

This study has limits. A limited number of patients were selected and evaluated; most of them (8) had a congenitally missing lateral incisor [40]. The intraoral scans were taken by two operators (although experienced and calibrated) at different times, with different environment conditions (room temperature, light, and more). Moreover, the assessment of tissue stability was only possible from the delivery of the final crown, which was used as a reference for the overlapping of 3D models; in this way, an evaluation of the tissues dynamics during provisionalization, and immediately following placement of the implant, was not possible. The only possible solution to evaluate soft tissues stability in the first 3 months after implant placement would be the use of the provisional restorations as references for the overlapping. In fact, the adjacent (natural) teeth cannot be used as references: they may be subject to movements, and these changes may render the overlapping of digital images rather inaccurate, jeopardizing the final 3D evaluation. However, the use of provisional restorations as references has limits: soft tissues are subjected to some kind of edema immediately after surgery, and this may introduce a bias in the study. Moreover, only modifications in a limited timeframe (3 months) can be registered, if provisional restorations are used as references for the overlapping procedures. It is very important to select proper landmarks for the overlapping of 3D models: these landmarks/reference points should be identified on the implant-supported restorations only, and not on the adjacent (natural) teeth. A possible solution for future studies should be the identification of two different timeframes, with a short-term evaluation of soft tissue stability during the provisionalization (first scan, S1, two weeks after implant placement; second scan, S2, 3 months later, before replacing the provisional with the final restoration) and then a long-term evaluation of soft tissues stability after the placement of final restoration (third scan, S3, immediately after the final restoration is placed; fourth scan, S4, 2 years later). Finally, the procedure for the overlapping of digital images is not easy, as it requires experience with the use of reverse-engineering software.

Beyond these considerations, the new method presented in this paper allows a detailed quantitative 3D evaluation of peri-implant soft tissue modifications along time. This could help to evaluate treatment results in the aesthetic areas of the anterior maxilla and therefore to identify the best treatment modalities (immediate versus early versus conventional implant placement) in different clinical situations, for achieving and maintaining aesthetic success in the long-term.

## 5. Conclusions

In the present study, we have introduced a new 3D method for the quantitative evaluation of soft tissue stability around single implants inserted to replace failing/nonrestorable and missing lateral incisors. This method is based on the overlapping of 3D models obtained from intraoral scans of the same patient taken at different times (at the delivery of the final crown and 2 years later). Within the limits of this study (limited number of patients treated and scans taken by different operators at different time) the new method introduced here can help to evaluate treatment results in the aesthetic areas of the anterior maxilla; therefore it could help to identify the best treatment modalities (immediate versus early versus conventional implant placement) for achieving and maintaining aesthetic success.

## Figures and Tables

**Table 1:** Soft tissue contraction around single implants inserted to replace failing/nonrestorable (*test* group: immediate implant placement in postextraction socket) and missing (*control* group: conventional implant placement in healed ridge) lateral incisors. The assessment of soft tissue contraction was performed via calculation of the Euclidean surface distances between the 3D models, after the superimposition of S2 on S1, in mm, over a 2-year period.

Immediate implant placement in postextraction sockets (*test* group)	Conventional implant placement in healed ridges (*control* group)
0.024	0.091
0.048	0.044
0.09	0.025
0.065	0.038
0.051	0.022
0.042	0.037
0.028	0.025
0.044	0.028
0.099	0.033
0.079	0.028

Overall: 0.057 (±0.025)	Overall: 0.037 (±0.020)

## References

[1] Chen S. T., Buser D. (2014). Esthetic outcomes following immediate and early implant placement in the anterior maxilla—a systematic review. *The International Journal of Oral & Maxillofacial Implants*.

[2] Mangano F., Mangano C., Ricci M., Sammons R. L., Shibli J. A., Piattelli A. (2012). Single-tooth Morse taper connection implants placed in fresh extraction sockets of the anterior maxilla: an aesthetic evaluation. *Clinical Oral Implants Research*.

[3] Kan J. Y. K., Rungcharassaeng K., Umezu K., Kois J. C. (2003). Dimensions of peri-implant mucosa: an evaluation of maxillary anterior single implants in humans. *Journal of Periodontology*.

[4] Buser D., Chappuis V., Bornstein M. M., Wittneben J.-G., Frei M., Belser U. C. (2013). Long-term stability of contour augmentation with early implant placement following single tooth extraction in the esthetic zone: a prospective, cross-sectional study in 41 patients with a 5- to 9-year follow-up. *Journal of Periodontology*.

[5] Covani U., Ricci M., Bozzolo G., Mangano F., Zini A., Barone A. (2011). Analysis of the pattern of the alveolar ridge remodelling following single tooth extraction. *Clinical Oral Implants Research*.

[6] Juodzbalys G., Wang H.-L. (2007). Soft and hard tissue assessment of immediate implant placement: a case series. *Clinical Oral Implants Research*.

[7] Tan W. L., Wong T. L. T., Wong M. C. M., Lang N. P. (2012). A systematic review of post-extractional alveolar hard and soft tissue dimensional changes in humans. *Clinical Oral Implants Research*.

[8] Araújo M. G., Silva C. O., Misawa M., Sukekava F. (2015). Alveolar socket healing: what can we learn?. *Periodontology 2000*.

[9] Araujo M. G., Lindhe J. (2005). Dimensional ridge alterations following tooth extraction. An experimental study in the dog. *Journal of Clinical Periodontology*.

[10] Araújo M. G., Lindhe J. (2009). Ridge alterations following tooth extraction with and without flap elevation: an experimental study in the dog. *Clinical Oral Implants Research*.

[11] Fickl S., Zuhr O., Wachtel H., Kebschull M., Hürzeler M. B. (2009). Hard tissue alterations after socket preservation with additional buccal overbuilding: a study in the beagle dog. *Journal of Clinical Periodontology*.

[12] Blanco J., Mareque S., Linares A., Munoz F. (2011). Vertical and horizontal ridge alterations after tooth extraction in the dog: flap vs. flapless surgery. *Clinical Oral Implants Research*.

[13] Botticelli D., Berglundh T., Lindhe J. (2004). Hard-tissue alterations following immediate implant placement in extraction sites. *Journal of Clinical Periodontology*.

[14] Chappuis V., Engel O., Reyes M., Shahim K., Nolte L.-P., Buser D. (2013). Ridge alterations post-extraction in the esthetic zone: a 3D analysis with CBCT. *Journal of Dental Research*.

[15] Farmer M., Darby I. (2014). Ridge dimensional changes following single-tooth extraction in the aesthetic zone. *Clinical Oral Implants Research*.

[16] Araújo M. G., da Silva J. C. C., de Mendonça A. F., Lindhe J. (2015). Ridge alterations following grafting of fresh extraction sockets in man: a randomized clinical trial. *Clinical Oral Implants Research*.

[17] Romanos G. E. (2015). Tissue preservation strategies for fostering long-term soft and hard tissue stability. *The International Journal of Periodontics & Restorative Dentistry*.

[18] Thoma D. S., Mühlemann S., Jung R. E. (2014). Critical soft-tissue dimensions with dental implants and treatment concepts. *Periodontology 2000*.

[19] Jemt T. (1997). Regeneration of gingival papillae after single-implant treatment. *The International Journal of Periodontics and Restorative Dentistry*.

[20] Meijer H. J. A., Stellingsma K., Meijndert L., Raghoebar G. M. (2005). A new index for rating aesthetics of implant-supported single crowns and adjacent soft tissues—the implant crown aesthetic index: a pilot study on validation of a new index. *Clinical Oral Implants Research*.

[21] Fürhauser R., Florescu D., Benesch T., Haas R., Mailath G., Watzek G. (2005). Evaluation of soft tissue around single-tooth implant crowns: the pink esthetic score. *Clinical Oral Implants Research*.

[22] Belser U. C., Grütter L., Vailati F., Bornstein M. M., Weber H.-P., Buser D. (2009). Outcome evaluation of early placed maxillary anterior single-tooth implants using objective esthetic criteria: a cross-sectional, retrospective study in 45 patients with a 2- to 4-year follow-up using pink and white esthetic scores. *Journal of Periodontology*.

[23] Hosseini M., Gotfredsen K. (2012). A feasible, aesthetic quality evaluation of implant-supported single crowns: an analysis of validity and reliability. *Clinical Oral Implants Research*.

[24] Vilhjálmsson V. H., Klock K. S., Størksen K., Bårdsen A. (2011). Aesthetics of implant-supported single anterior maxillary crowns evaluated by objective indices and participants' perceptions. *Clinical Oral Implants Research*.

[25] Mangano F. G., Mangano C., Ricci M., Sammons R. L., Shibli J. A., Piattelli A. (2013). Esthetic evaluation of single-tooth morse taper connection implants placed in fresh extraction sockets or healed sites. *Journal of Oral Implantology*.

[26] Raes F., Cosyn J., De Bruyn H. (2013). Clinical, aesthetic, and patient-related outcome of immediately loaded single implants in the anterior maxilla: A Prospective Study in Extraction Sockets, Healed Ridges, and Grafted Sites. *Clinical Implant Dentistry and Related Research*.

[27] Cosyn J., Eghbali A., Hanselaer L. (2013). Four modalities of single implant treatment in the anterior maxilla: a clinical, radiographic, and aesthetic evaluation. *Clinical Implant Dentistry and Related Research*.

[28] Benic G. I., Wolleb K., Sancho-Puchades M., Hämmerle C. H. F. (2012). Systematic review of parameters and methods for the professional assessment of aesthetics in dental implant research. *Journal of Clinical Periodontology*.

[29] Chappuis V., Engel O., Shahim K., Reyes M., Katsaros C., Buser D. (2015). Soft tissue alterations in esthetic postextraction sites: a 3-dimensional analysis. *Journal of Dental Research*.

[30] Zimmermann M., Mehl A., Mörmann W. H., Reich S. (2015). Intraoral scanning systems—a current overview. *International Journal of Computerized Dentistry*.

[31] Ting-Shu S., Jian S. (2015). Intraoral digital impression technique: a review. *Journal of Prosthodontics*.

[32] Cevidanes L. H. C., Oliveira A. E. F., Grauer D., Styner M., Proffit W. R. (2011). Clinical application of 3D imaging for assessment of treatment outcomes. *Seminars in Orthodontics*.

[33] Gkantidis N., Schauseil M., Pazera P., Zorkun B., Katsaros C., Ludwig B. (2015). Evaluation of 3-dimensional superimposition techniques on various skeletal structures of the head using surface models. *PLoS ONE*.

[34] Chrcanovic B. R., Albrektsson T., Wennerberg A. (2015). Smoking and dental implants: a systematic review and meta-analysis. *Journal of Dentistry*.

[35] Luongo G., Lenzi C., Raes F., Eccellente T., Ortolani M., Mangano C. (2014). Immediate functional loading of single implants: a 1-year interim report of a 5-year prospective multicentre study. *European Journal of Oral Implantology*.

[36] Lee S.-Y., Yang D.-J., Yeo S., An H.-W., Ryoo K. H., Park K.-B. (2012). The cytocompatibility and osseointegration of the Ti implants with XPEED® surfaces. *Clinical Oral Implants Research*.

[37] Huynh-Ba G., Pjetursson B. E., Sanz M. (2010). Analysis of the socket bone wall dimensions in the upper maxilla in relation to immediate implant placement. *Clinical Oral Implants Research*.

[38] Januário A. L., Duarte W. R., Barriviera M., Mesti J. C., Araújo M. G., Lindhe J. (2011). Dimension of the facial bone wall in the anterior maxilla: a cone-beam computed tomography study. *Clinical Oral Implants Research*.

[39] Nisapakultorn K., Suphanantachat S., Silkosessak O., Rattanamongkolgul S. (2010). Factors affecting soft tissue level around anterior maxillary single-tooth implants. *Clinical Oral Implants Research*.

[40] Mangano C., Levrini L., Mangano A., Mangano F., MacChi A., Caprioglio A. (2014). Esthetic evaluation of implants placed after orthodontic treatment in patients with congenitally missing lateral incisors. *Journal of Esthetic and Restorative Dentistry*.

